# Cerebrospinal fluid sclerostin levels in the early Alzheimer's disease stages

**DOI:** 10.1002/dad2.70297

**Published:** 2026-03-11

**Authors:** Manuela Dicarlo, Patrizia Pignataro, Daniele Urso, Chiara Zecca, Maria Teresa Dell'Abate, Francesco Borlizzi, Valentina Gnoni, Alessia Giugno, Angela Oranger, Graziana Colaianni, Roberta Zerlotin, Clelia Suriano, Silvia Colucci, Giancarlo Logroscino, Maria Grano

**Affiliations:** ^1^ Department of Precision and Regenerative Medicine and Ionian Area (DiMePRe‐J) University of Bari “A. Moro,” Bari Italy; ^2^ Department of Translational Biomedicine and Neuroscience (DiBraiN) University of Bari “A. Moro,” Bari Italy; ^3^ Center for Neurodegenerative Diseases and the Aging Brain University of Bari “A. Moro” at “Pia Fondazione Card G. Panico” Hospital Tricase Italy

**Keywords:** Alzheimer's disease, amyloid beta, dementia, mild cognitive impairment, neurodegeneration, phosphorylated tau, sclerostin, total tau

## Abstract

**INTRODUCTION:**

Sclerostin, a negative regulator of bone formation, has been involved in memory impairment in Alzheimer's disease (AD) mouse models and is increased in elderly people at risk of AD. Here, we investigated sclerostin's role across the clinical stages of AD.

**METHODS:**

We evaluated cerebrospinal fluid (CSF) sclerostin levels in patients with dementia due to AD, mild cognitive impairment, and subjective memory complaints, biologically characterized via the amyloid/tau/neurodegeneration classification. Results were correlated with AD biomarkers, amyloid beta (Aβ) 42, phosphorylated tau (p‐tau), and total tau (t‐tau), and clinical parameters of dementia severity.

**RESULTS:**

CSF sclerostin increased in patients with dementia due to AD and correlated negatively with Aβ42 and positively with p‐tau, t‐tau, and dementia severity.

**DISCUSSION:**

The association of CSF sclerostin with Aβ42, tau pathology, and dementia severity in the early disease stages is of great clinical relevance for the identification of sclerostin as a promising biomarker in early AD stages.

## Highlights

- We studied sclerostin levels in patients with Alzheimer's disease (AD) who had been biologically classified.
- Cerebrospinal fluid (CSF) sclerostin was increased in patients with AD.
- CSF sclerostin correlated with amyloid beta, phosphorylated tau, total tau, and dementia severity.
- Sclerostin could be a biomarker in the early phase of AD.

## 1 BACKGROUND

Alzheimer's disease (AD) is a neurodegenerative disorder primarily characterized by progressive decline in cognitive function and behavioral changes.^1^

AD is the most common cause of dementia (≈ 60%–70% of all dementia cases) and its prevalence is expected to grow to > 150 million globally by 2050.^1^, ^2^ While biomarker‐based diagnostic frameworks are now well established, several crucial aspects, such as the underlying causes of the disease and its associated biological processes, remain under intense investigation.^3^ Because pathological changes in AD begin many years before clinical symptoms emerge, and current treatments show limited efficacy in later stages, it is essential to study disease mechanisms and biomarkers throughout the entire disease continuum, from preclinical to clinical stages.^4^

In recent years, evidence has emerged suggesting that extra‐cerebral factors may contribute to AD pathogenesis and progression.^5^ The skeletal system, besides its mechanical and protective functions, displays an endocrine activity due to the production of several factors (osteokines) which are released in the circulation to target other organs including the brain.^6^ An association between bone disorders, such as osteoporosis, and AD has been frequently reported in epidemiological studies.^7^ These findings support the emerging concept of bone–brain crosstalk and the potential involvement of osteokines in AD pathogenesis, as highlighted by evidence from both animal and human studies.^8^ Osteocalcin, lipocalin‐2, fibroblast growth factor 23, osteoprotegerin, and osteopontin have been investigated for their involvement in cognitive functions and AD pathogenesis.^9^, ^10^, ^11^, ^12^, ^13^, ^14^

Very recently, sclerostin, an osteocyte‐derived glycoprotein that blocks osteoblast proliferation, differentiation, and activity, has gained interest as bone‐derived hormone involved in AD brain dysfunctions in mice.^15^ Indeed, it has been demonstrated that sclerostin can cross the blood–brain barrier and accelerate cognitive impairment by increasing amyloid beta (Aβ) production in mouse models of AD.^15^ However, the existing literature on human cohorts is limited to only two studies that did not focus on AD staging.^15^, ^16^

Therefore, in this study, we explored sclerostin's role in the AD continuum and its possible use as an AD biomarker by recruiting a large cohort of patients with dementia due to AD, mild cognitive impairment (MCI), and subjective memory complaints (SMC) biologically characterized according to the amyloid/tau/neurodegeneration (ATN) scheme of the National Institute on Aging–Alzheimer's Association (NIA‐AA).^17^ First, cerebrospinal fluid (CSF) sclerostin levels were compared between patients with dementia due to AD and those with MCI and SMC. Then, we correlated CSF sclerostin levels with fluid AD biomarkers, CSF Aβ42, phosphorylated tau (p‐tau), and total tau (t‐tau), and clinical parameters of dementia severity, that is, Clinical Dementia Rating Scale Global score (CDR‐GS) and CDR Sum of Boxes (CDR‐SOB).

## 2 METHODS

### 2.1 Participants

The cohort of this study consisted of 146 participants, referred to the Center for Neurodegenerative Diseases and the Aging Brain of the University of Study of Bari “Aldo Moro” at Pia Fondazione “Card. Panico” Hospital (Tricase, Italy). All participants underwent a standardized multidisciplinary protocol including neurological and neuropsychological examination, nutritional assessment, 3T magnetic resonance imaging, routine laboratory assessment, and lumbar puncture for the analysis of CSF biomarkers. The neuropsychological examination comprised 18 psychometric tests including the Mini‐Mental State Examination (MMSE) used as a global cognitive screening measure.^18^ Dementia severity and functional status were assessed using the CDR‐GS and CDR‐SOB.

Participants were classified into three groups based on established clinical criteria. Patients with dementia due to AD (*n* = 82) met the Diagnostic and Statistical Manual of Mental Disorders, 5th edition (DSM‐V)^19^ and the NIA‐AA 2024 clinical criteria.^17^ Dementia stage was defined by a CDR‐GS ≥ 1.

Participants with MCI (*n* = 44) met the following criteria: (1) cognitive concern that reflect a change in cognition reported by patient or informant or clinician, (2) impairment in one or more cognitive domains (i.e., memory, executive function, attention, visuospatial abilities, and language) documented by neuropsychological testing, (3) normal functional activities as derived from the CDR and the Functional Activities Questionnaire, and (4) absence of dementia (DSM‐V).

Participants with SMC (*n* = 20) were defined by: (1) self‐experienced persistent decline in memory and cognitive capacity compared to a previously normal status and unrelated to an acute event; and (2) normal age‐, sex‐, and education‐adjusted performance on standardized cognitive tests that are used for MCI or prodromal AD classification.^20^ The patients included in the SMC group had CDR‐GS = 0.

Biological classification was performed according to the ATN framework.^17^ All participants underwent lumbar puncture for CSF biomarker assessment. Participants in the MCI and dementia due to AD group showed biomarker evidence of AD pathology and were therefore classified as biologically positive for AD.^17^

All SMC participants had a negative AD biomarker profile (A–/T–/N–) according to the ATN classification.^17^

Blood–brain barrier integrity was also assessed by calculating the CSF/serum albumin quotient (Q‐Alb), obtained from albumin concentrations measured during routine chemical–physical examination of paired CSF and serum samples.

This study was approved by the local ethical committee (ASL Lecce verbale No. 6, May 25, 2017), according to the Declaration of Helsinki.^21^ All participants provided their written informed consent and were enrolled in the study only on the basis of their clinical diagnosis, without age, sex, and race discriminations. Patients were carefully recruited to ensure sex balance and to avoid racial discrimination.

### 2.2 CSF collection and storage

Patient CSF samples were collected via lumbar puncture according to standard procedures. Samples were centrifuged at room temperature for 10 minutes at 2000 g, aliquoted, and immediately stored at −80°C until their use, according to the international biomarkers recommendations.^22^

### 2.3 CSF AD biomarkers analysis

CSF concentrations of Aβ42, t‐tau, and p‐tau181 were quantified in patient samples by chemiluminescent immunoassay (CLEIA; Lumipulse G amyloid β 1‐42, Lumipulse G Total Tau, Lumipulse G pTau181, Fujirebio Europe N.V.) on a fully automatic platform (Lumipulse G600II, Fujirebio Europe N.V.). All the assays were executed according to manufacturer's instructions.

**RESEARCH IN CONTEXT**

1. **Systematic review**: The authors reviewed the literature using traditional sources (e.g., PubMed). Sclerostin is an osteocyte‐derived factor that has been very recently explored in the context of Alzheimer's disease (AD) for its ability to cross the blood–brain barrier increasing amyloid beta (Aβ) deposition in AD mouse models. Very limited studies on humans did not account for AD staging, thus investigations in the clinical stages of AD are needed.
2. **Interpretation**: We enrolled patients biologically defined according to the amyloid/tau/neurodegeneration criteria and we found increased sclerostin levels in the cerebrospinal fluid (CSF) of patients with AD, remarkably in those with mild dementia. Moreover, we observed correlations with CSF Aβ42, phosphorylated and total tau, and dementia severity.
3. **Future directions**: Additional studies investigating serum and/or plasma sclerostin levels in the continuum of AD pathology will further elucidate the clinical relevance of the osteokine.

The following cut‐off values were considered for the correct interpretation of CSF biomarker results: Aβ42 > 599 pg/mL, t‐tau < 342 pg/mL, and p‐tau181 < 57 pg/mL. In accordance with the diagnostic criteria for AD,^23^ a CSF biomarker profile was considered indicative of AD if the CSF Aβ42 value was below the cut‐off, in conjunction with t‐tau and/or p‐tau181 values above the cut‐off. In selected cases with borderline amyloid values, the p‐tau/Aβ42 ratio was additionally considered to support amyloid status interpretation.^24^

### 2.4 CSF sclerostin assay

Sclerostin levels were measured in CSF patient samples using the Human SOST/Sclerostin Quantikine enzyme‐linked immunosorbent assay (ELISA) kit (DSST00, R&D Systems, Inc.). The minimum detectable dose (MDD) was 3.8 pg/mL with a measurement range of 31.3 to 2000 pg/mL. All patient CSF samples and standard dilutions were run in duplicate. The plate absorbance was read at 450 nm in a plate reader (Eon, BioTek). Optical imperfections in the plate were corrected by subtracting readings at 540 nm from the readings at 450 nm. Results were reported in pg/mL.

### 2.5 Statistical analysis

Statistical analyses were conducted using GraphPad Prism statistical software (Version 9.5.0; GraphPad Software) and JASP free software (Version 0.19.3; JASP Team), with a significance threshold of *p* < 0.05. The normality of continuous variables was initially assessed using the Shapiro–Wilk test. Two group comparisons were evaluated by two‐tailed unpaired Student test or Mann–Whitney test. Analysis of variance or Kruskal–Wallis and their post hoc with multiple comparison corrections (Tukey and Dunn, respectively) were used for three‐group comparisons. Sex differences were analyzed using chi‐squared tests. Correlation analyses were performed by the Spearman correlation coefficient test. Partial correlation analyses with age and sex as covariates were performed using the JASP software. In the absence of a clinically validated cutoff, for some subgroup comparisons, patients with MCI and AD were stratified based on their CSF sclerostin levels as low (under the median value) and high (over the median value). This approach is commonly used in exploratory analyses, and using the median ensures an approximately equal distribution of participants in each group, enhancing statistical power and estimate stability.

## 3 RESULTS

### 3.1 Participant characteristics

Table 1 provides participant demographics and results from biomarker measurement and clinical evaluations. The study cohort that consisted of patients with SMC, MCI, and dementia due to AD, was sex balanced (*p* = 0.367), while the mean age of patients with dementia due to AD was higher compared to SMC (*p* = 0.006). The integrity of the blood–brain barrier, evaluated by CSF/serum albumin quotient (Q‐Alb), did not differ among groups.

**TABLE 1:** Participant demographic and clinical data.

Patient characteristics	SMC *n* = 20	MCI *n* = 44	AD *n* = 82	*p*
**Demographics**				
Age (years)				
Mean ± SD	60.35 ± 10.97	63.64 ± 10.74	67.51 ± 7.81	**0.006** SMC vs. AD
Sex, number (%)				
Women	9 (45)	25 (57)	51 (62)	
Men	11 (55)	19 (43)	31 (38)	0.367 vs. women
**Marker of blood–CSF barrier integrity**				
Q‐Alb				
Median (Q1–Q3)	7.95 (5.76–10.60)	5.50 (4.25–7.65)	6.10 (4.80–8.20)	
**Biological AD biomarkers**				
CSF Aβ42 (pg/mL)				
Median (Q1–Q3)	895.5 (829.6–1182)	511.5 (402.4–653.8)	485.5 (366.4–569)	**< 0.0001** SMC vs. MCI and SMC vs. AD
CSF p‐tau (pg/mL)				
Median (Q1–Q3)	28.86 (22.63–37.2)	47.54 (25.3–81.98)	82.6 (52.96–112.7)	**0.0120** SMC vs. MCI; **< 0.0001** SMC vs. AD; **0.0013** MCI vs. AD
CSF t‐tau (pg/mL)				
Median (Q1–Q3)	186.5 (141.7–235.8)	345.5 (181.5–582.3)	622 (419.7–845.4)	**0.0106** SMC vs. MCI; **< 0.0001** SMC vs. AD; **0.0005** MCI vs. AD
CSF p‐tau (pg/mL)/ CSF Aβ42 (pg/mL)				
Median (Q1–Q3)	0.03 (0.03–0.04)	0.11 (0.04–0.18)	0.18 (0.11–0.28)	**0.0002** SMC vs. MCI; **< 0.0001** SMC vs. AD; **0.002** MCI vs. AD
**Clinical parameters of dementia severity**				
CDR‐SOB				
Median (Q1–Q3)	0 (0–0.5)	2.5 (1.5–3.5)	4.5 (3.5–7.5)	**0.0073** SMC vs. MCI; **< 0.0001** SMC vs. AD and MCI vs. AD
CDR‐GS				
Median (Q1–Q3)	0 (0–0)	0.5 (0.5–0.5)	1 (1–1)	**0.0012** SMC vs. MCI; ** < 0.0001** SMC vs. AD and MCI vs. AD

*Notes*: Bold values highlight statistically significant differences among patient groups (analysis of variance Tukey test and Kruskal–Wallis–Dunn test, *p* < 0.05, for continuous data; Pearson chi squared test, *p *< 0.05, for sex).

Abbreviations: AD, Alzheimer's disease; Aβ, amyloid beta; CDR‐GS, Clinical Dementia Rating Global Score; CDR‐SOB, Clinical Dementia Rating Scale Sum of Boxes; CSF, cerebrospinal fluid; MCI, mild cognitive impairment; Q1, lower quartile; Q2, upper quartile; Q‐Alb, CSF/serum albumin quotient; p‐tau, phosphorylated tau; SD, standard deviation; SMC, subjective memory complaints; t‐tau, total tau.

Following the research framework,^17^ patients with dementia due to AD had lower levels of Aβ42 (*p* < 0.0001 vs. SMC) and higher concentrations of t‐tau (*p* < 0.0001 vs. SMC and *p* = 0.0005 vs. MCI) and p‐tau (*p* < 0.0001 vs. SMC and *p* = 0.0013 vs. MCI) in the CSF. Moreover, patients with dementia due to AD showed significantly higher CDR‐GS and CDR‐SOB scores compared to patients with MCI and SMC (*p* < 0.0001 vs. SMC and MCI).

### 3.2 CSF sclerostin levels in patients with SMC, MCI, and AD

Table 2 summarizes the results of this analysis and the comparison among groups and subgroups of patients.

**TABLE 2:** CSF sclerostin levels in patients with SMC, MCI, and AD.

	Groups		
	All patients	Women	Men
**SMC (reference)**			
Mean value ± SD	0 ± 0	0 ± 0	0 ± 0
Median (Q1–Q3)	0 (0–0)	0 (0–0)	0 (0–0)
Min	0	0	0
Max	0	0	0
**MCI**			
Mean value ± SD	2.31 ± 6.89	3.54 ± 8.87	0.68 ± 1.86
Median (Q1–Q3)	0 (0–0)	0 (0–0.372)	0 (0–0)
Min	0	0	0
Max	35.23	35.23	6.11
*p* value	0.356 vs. SMC	0.563 vs. SMC	> 0.999 vs. SMC 0.438 vs. women
**AD**			
Mean value ± SD	6.85 ± 15.24	7.20 ± 17.54	6.28 ± 10.67
Median (Q1–Q3)	0 (0–7.77)	0 (0–8.07)	0.93 (0–7.66)
Min	0	0	0
Max	101.4	101.4	46.07
*p* value	**0.001 vs. SMC** **0.032 vs. MCI**	0.100 vs. SMC 0.877 vs. MCI	**0.005 vs. SMC** **0.0.17 vs. MCI** 0.293 vs. women

*Notes*: Bold values highlight statistically significant differences with respect to the SMC patients (Kruskal–Wallis–Dunn test, *p* < 0.05) and between men and women (two‐tailed unpaired Mann–Whitney test, *p* < 0.05).

Abbreviations: AD, Alzheimer's disease; CSF, cerebrospinal fluid; Max, maximum value; Min, minimum value; MCI, mild cognitive impairment; Q1, lower quartile; Q3, upper quartile; SD, standard deviation; SMC, subjective memory complaints.

**FIGURE 1:**
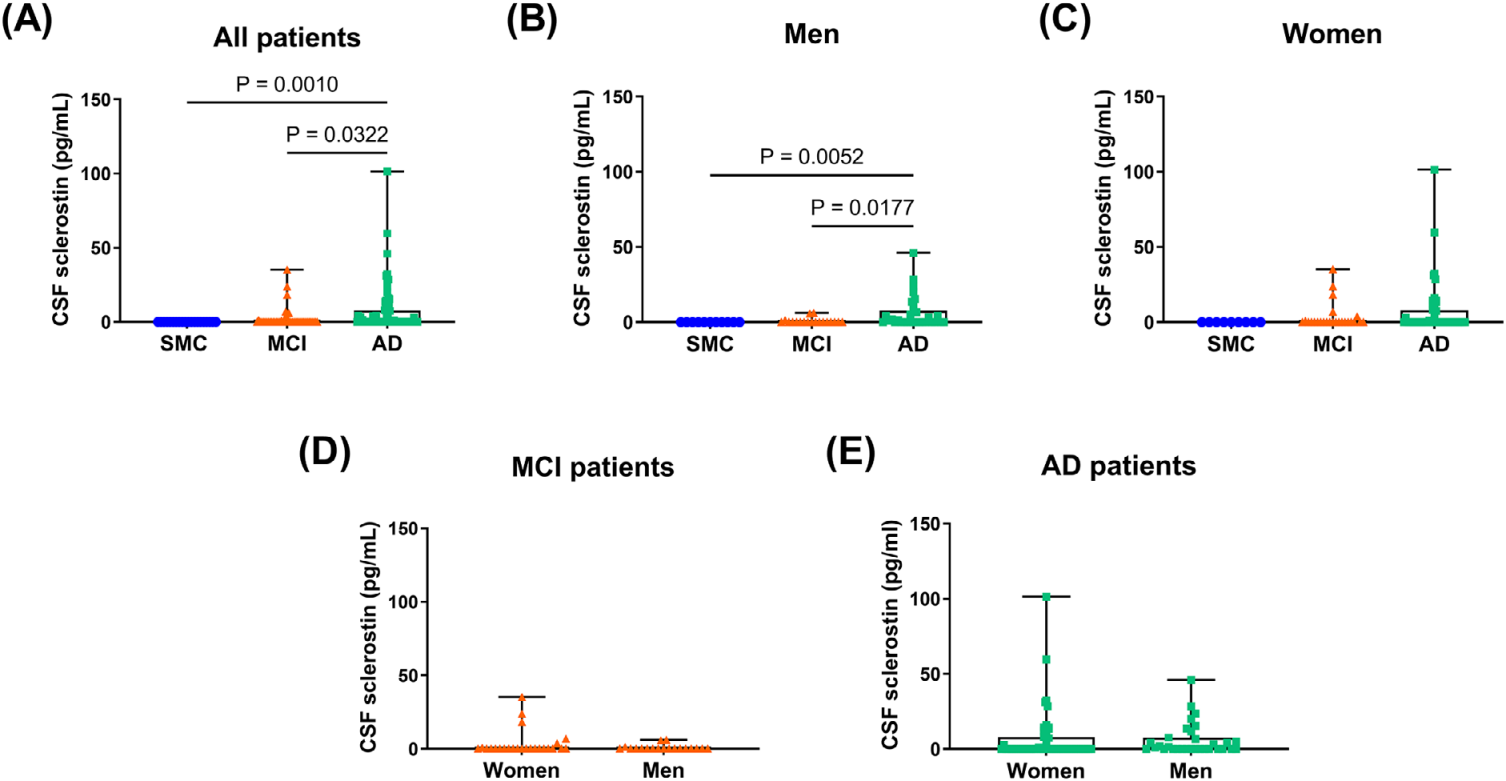
Comparison of CSF sclerostin levels in patients with SMC, MCI, and dementia due to AD. A, CSF sclerostin levels were significantly increased in patients with dementia due to AD compared to SMC and MCI. B, The same trend was mantained in male patients, while (C) no difference was found in the female subgroup. D, No sex differences were noted in MCI and (E) AD subgroup analysis. Data are presented as box‐and‐whisker with median and interquartile ranges, from max to min, with all data points shown. Horizontal bars show significant differences among groups (Kruskal–Wallis test/Dunn multiple comparison test or Mann–Whitney test, *p* < 0.05). AD, Alzheimer's disease; CSF, cerebrospinal fluid; MCI, mild cognitive impairment; SMC, subjective memory complaints.

CSF sclerostin levels were significantly increased in patients with dementia due to AD compared to both MCI (*p* = 0.0322) and SMC (*p* = 0.0010). Sclerostin was undetectable in all patients with SMC, while more samples from patients with MCI and AD showed detectable sclerostin levels (Figure 1A). This pattern was evident in male patients, with significantly higher CSF sclerostin levels in AD compared to MCI and SMC (*p* = 0.0177 and *p* = 0.0052, respectively; Figure 1B), while no significant differences were observed among females (Figure 1C).

**FIGURE 2:**
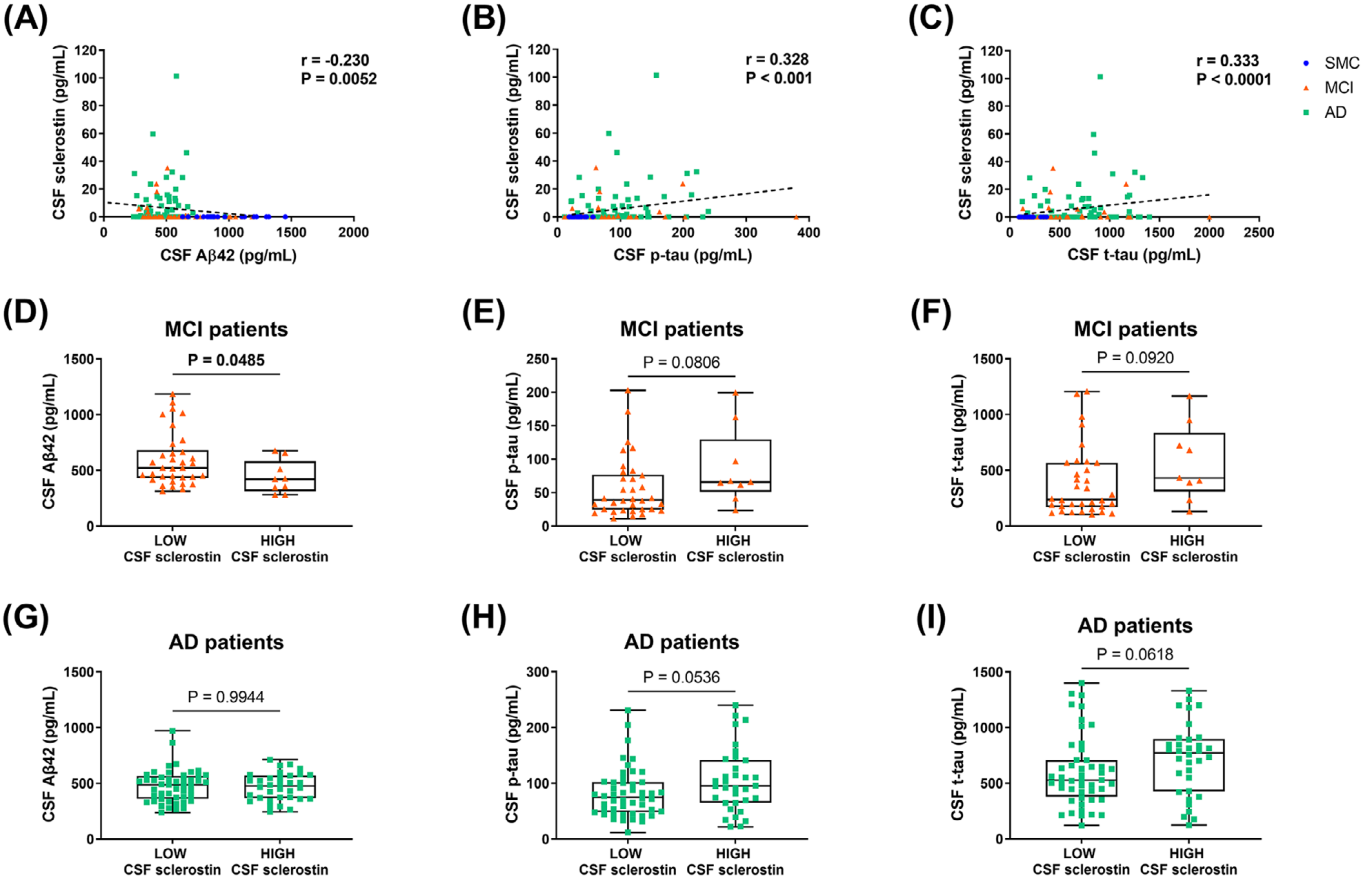
Correlation of CSF sclerostin levels and AD biomarkers. A, Sclerostin levels correlated negatively with CSF Aβ42 and positively with (B) p‐tau, and (C) t‐tau. D, Significantly lower CSF Aβ42 levels were observed in MCI patients with high CSF sclerostin, while trends of increase were found for (E) p‐tau and (F) t‐tau level compared to MCI patients with low CSF sclerostin. G, No difference was noted for Aβ42 levels between patients with dementia due to AD with high and low CSF sclerostin levels, while trends of increase were observed for (H) p‐tau and (I) t‐tau levels. In correlation plots, the dotted lines represent Spearman linear regressions (*r* and *p* values as indicated). For two‐group comparison, data are presented as box‐and‐whisker with median and interquartile ranges, from max to min, with all data points shown. Horizontal bars show the statistical analysis between groups (Student test or Mann–Whitney test, *p* < 0.05). Bold values highlight statistically significant results. Aβ, amyloid beta; AD, Alzheimer's disease; CSF, cerebrospinal fluid; MCI, mild cognitive impairment; p‐tau, phosphorylated tau; SMC, subjective memory complaints; t‐tau, total tau.

Sex‐stratified analyses in patients with MCI and AD showed no significant differences between women and men (Figure 1D and E).

### 3.3 Correlation of CSF sclerostin and AD biomarkers

First, we evaluated the association between CSF sclerostin levels and age. We found a positive correlation in overall patients (*r* = 0.243, *p* = 0.0032; Figure S1A in supporting information) and in male patients (*r* = 0.302, *p* = 0.018; Figure S1B), while a positive trend was observed in females (*r* = 0.208, *p* = 0.056; Figure S1C).

For blood–brain barrier integrity, we evaluated the correlation with CSF/serum albumin quotient (Q‐Alb) values, and we found a positive correlation with CSF sclerostin levels (*r* = 0.188, *p* = 0.023; Figure S2A in supporting information). This correlation persisted when corrected for age and sex (*r* = 0.201, *p* = 0.015). In addition, we observed no differences in Q‐Alb values between patients with MCI with low and high CSF sclerostin (Figure S2B), while higher Q‐Alb values were associated with higher CSF sclerostin levels in patients with AD (Figure S2C).

CSF sclerostin levels were then correlated with CSF AD biomarkers (i.e., Aβ42, p‐tau, and t‐tau). Table 3 summarizes the results of this analysis including both uncorrected correlations and partial correlations adjusted for age and sex.

**TABLE 3:** Correlations between CSF sclerostin levels with AD biomarkers and clinical data.

	Uncorrected		Corrected for age and sex	
	*r*	*p*	*r*	*p*
CSF Aβ42	−0.230	**0.0052**	−0.215	**0.010**
CSF p‐tau	0.328	**< 0.001**	0.285	**<0.001**
CSF t‐tau	0.333	**< 0.0001**	0.291	**<0.001**
CDR‐GS	0.198	**0.0223**	0.119	0.156
CDR‐SOB	0.156	0.0900	0.130	0.163

*Notes*: Bold values highlight statistically significant correlations (Spearman correlation coefficient test; *r*, Spearman correlation coefficient). *r* and *p* uncorrected correspond to Spearman correlation analysis, *r* and *p* corrected correspond to partial correlation corrected for age and sex.

Abbreviations: Aβ, amyloid beta; AD, Alzheimer's disease; CDR‐GR, Clinical Dementia Rating Scale Global Score; CDR‐SOB, Clinical Dementia Rating Scale Sum of Boxes; CSF, cerebrospinal fluid; p‐tau, phosphorylated tau; t‐tau, total tau.

We found a negative correlation between CSF sclerostin levels and Aβ42 levels (*r* = –0.230, *p* = 0.0052; Figure 2A), and a positive correlation with p‐tau (*r* = 0.328, *p* < 0.001) and t‐tau (*r* = 0.333, *p* < 0.0001) in the CSF in overall patients (Figure 2B and C).

Partial correlation analysis evidenced that the correlation between CSF sclerostin and Aβ42, p‐tau, and t‐tau remained significant when corrected for age and sex (*r* = –0.215 and *p* = 0.010 for Aβ42; *r* = 0.285 and *p* < 0.001 for p‐tau, and *r* = 0.291 and *p* < 0.001 for t‐tau). In addition, we observed significantly lower CSF Aβ42 levels (*p* = 0.0485; Figure 2D) and trends of increase in p‐tau (*p* = 0.0806; Figure 2E) and t‐tau levels (*p* = 0.0920; Figure 2F) in patients with MCI with high CSF sclerostin levels.

No difference was noted for Aβ42 levels between patients with AD with high and low CSF sclerostin levels (*p* = 0.9944; Figure 2G), while trends of increase were observed for p‐tau (*p* = 0.0536; Figure 2H) and t‐tau levels (*p* = 0.0618) in patients with AD with high CSF sclerostin levels (Figure 2I).

Similar results emerged stratifying patients by stage (MCI and dementia due to AD). We noticed a negative correlation between CSF sclerostin levels and Aβ42 levels (*r* = –0.322, *p* = 0.033; Figure S3A in supporting information), and positive trends with p‐tau (*r* = 0.240, *p* = 0.116) and t‐tau (*r* = 0.230, *p* = 0.133) in patients with MCI (Figure S3B and S3C). For patients with AD, we observed no correlation between CSF sclerostin levels and Aβ42 levels (*r* = 0.042, *p* = 0.708; Figure S4A in supporting information), and positive correlations with p‐tau (*r* = 0.236, *p* = 0.033) and t‐tau (*r* = 0.232, *p* = 0.036; Figure S4B and S4C).

### 3.4 Correlation of CSF sclerostin and clinical parameters of dementia severity

A correlation analysis was performed to evaluate the association of CSF sclerostin levels with CDR‐GS and CDR‐SOB.

The results of this analysis including both uncorrected correlations and partial correlations adjusted for age and sex are summarized in the lowest rows of Table 3.

**FIGURE 3:**
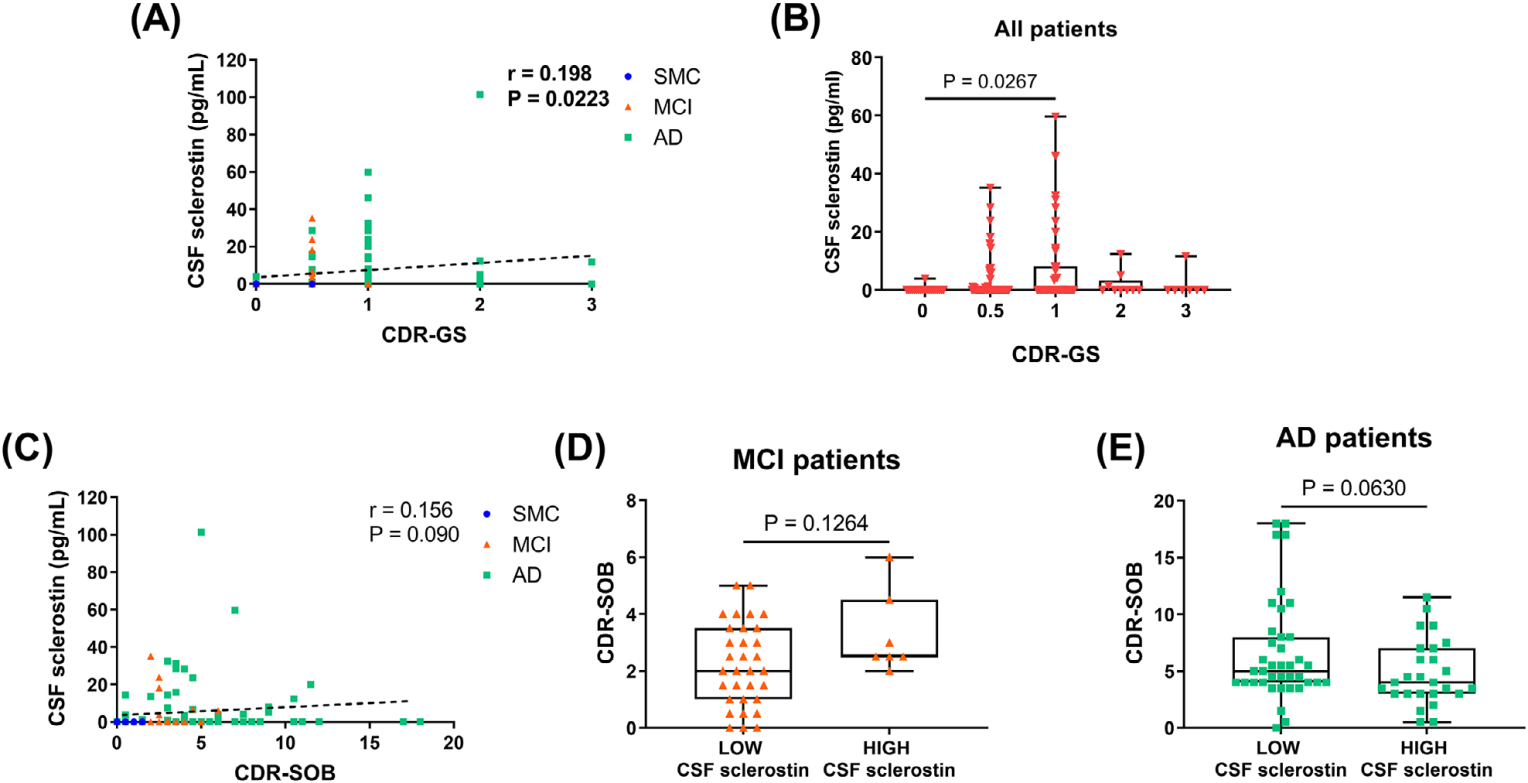
Correlation of CSF sclerostin levels and clinical parameters of dementia severity. A, CSF sclerostin positively correlated with CDR‐GS. B, CSF sclerostin levels were higher in patients with mild dementia (CDR‐GS = 1) compared to non‐demented patients (CDR‐GS = 0). C, A positive trend was observed between CSF sclerostin and CDR‐SOB. D, No differences were observed for CDR‐SOB in MCI and (E) patients with AD with high or low CSF sclerostin levels. In correlation plots, the dotted lines represent Spearman linear regressions (*r* and *p* values as indicated). For two‐group comparison, data are presented as box‐and‐whisker with median and interquartile ranges, from max to min, with all data points shown. Horizontal bars show the statistical analysis between groups (Student test or Mann–Whitney test, *p* < 0.05). Bold values highlight statistically significant results. AD, Alzheimer's disease; CSF, cerebrospinal fluid; CDR‐GS, Clinical Dementia Rating Scale Global Score; CDR‐SOB, Clinical Dementia Rating Scale Sum of Boxes; MCI, mild cognitive impairment; SMC, subjective memory complaints.

For CDR‐GS, we evidenced a positive correlation with CSF sclerostin levels (*r* = 0.198, *p* = 0.0223; Figure 3A). Furthermore, stratifying the study cohort based on their CDR‐GS (from 0 to 3), we noticed a significant increase in CSF sclerostin levels in patients with mild dementia (CDR‐GS = 1) compared to non‐demented patients (CDR‐GS = 0; *p* = 0.0267; Figure 3B).

For CDR‐SOB, we found a positive trend with CSF sclerostin levels (*r* = 0.156, *p* = 0.090; Figure 3C), while no differences were noticed in patients with MCI (Figure 3D) and AD (Figure 3E) with low and high CSF sclerostin levels. The correlation with both CDR‐GS and CDR‐SOB did not remain significant when corrected for age and sex (*r* = 0.119, *p* = 0.156 and *r* = 0.130, *p* = 0.163, respectively).

## 4 DISCUSSION

In the present study, we focused on the role of the osteokine sclerostin in the continuum of AD showing that CSF sclerostin levels significantly increased in patients with dementia due to AD, with the highest sclerostin concentrations detected in the early stages of clinical AD (i.e., patients with CDR‐GS = 1). Sclerostin levels were negatively correlated with Aβ42 and positively correlated with p‐tau and t‐tau. Positive trends were also evidenced between sclerostin and clinical parameters of dementia severity, that is, CDR‐GS and CDR‐SOB.

Sclerostin, an antagonist of the Wnt‐β catenin signaling pathway by its binding to the LRP5/6 receptor, is primarily secreted by mature osteocytes.^25^ As a negative regulator of bone formation, sclerostin acts on mature osteoblasts and their precursors, inhibiting both their activity and differentiation.^26^ Besides its effect on bone, the negative impact of osteocyte‐derived sclerostin on memory and synaptic plasticity in aged mice and in AD mouse models was recently shown,^15^ paving new ways toward extra‐skeletal roles of this factor previously confined to bone tissue.

Notably, at present only a few reports have investigated sclerostin involvement in AD pathogenesis in human cohorts.^15^, ^16^ The first study on this topic focused on cognitively normal older adults at high risk of AD, while the second, by Shi et al., considered clinically diagnosed patients with AD.^15^, ^16^ Different from previous literature, we measured sclerostin in the CSF and, to better evaluate the mechanistic link between sclerostin and AD, we adopted the research framework of ATN for patient selection, demonstrating, for the first time, that CSF sclerostin levels were increased in the continuum of AD. Similar trends were observed in male and female subgroup analysis with a slight difference for female patients with AD that did not show higher sclerostin levels compared to females with MCI and SMC. In addition, no difference was noted between female and male patients with MCI and AD; however, this finding may be partially explained by a lack of statistical power in the stratified analyses, despite the observation of several women with high CSF sclerostin concentrations. At present, there are no data on CSF in healthy subjects; however, it is known that higher serum sclerostin levels have been detected in healthy men compared to women.^27^ Sex‐dependent differences in circulating sclerostin levels are likely attributable to the complex interplay among skeletal mass, hormonal regulation, and life‐stage–specific factors such as menopause. Sex differences in sclerostin may partially mirror structural differences in the skeleton. Indeed, circulating sclerostin concentrations have been shown to correlate positively with bone size and bone mineral content, suggesting that higher levels may reflect skeletal volume rather than sex per se.^27^, ^28^ However, beyond skeletal mass, sex hormones exert a strong regulatory influence on sclerostin expression.^29^ For female sex steroid hormones, experimental and clinical evidence suggested an inhibitory role of estrogens on sclerostin expression; consequently, estrogen deficiency may contribute to reduced bone formation and accelerated bone loss in women.^30^, ^31^ Consistent with this mechanism, estrogen replacement therapy in postmenopausal women has been associated with reduced circulating sclerostin levels.^31^, ^32^ Menopause therefore represents a critical transition in skeletal biology and is accompanied by marked changes in sclerostin levels. Several studies have reported increased circulating sclerostin concentrations after menopause, consistent with the abrupt decline in estrogen levels and the subsequent uncoupling of bone remodeling.^31^ This increase may partially explain the attenuation or modification of sex‐related differences in sclerostin levels in older populations, such as our study cohort.

Furthermore, there may be additional factors, linked to AD pathogenesis, that interfered with CSF sclerostin levels in a sex‐specific manner and will require more in‐depth investigations. Indeed, there may be underlying biological differences, to such an extent that a discrepancy is observed compared to previously reported plasma sclerostin results.^16^ CSF sclerostin may reflect both central and peripheral mechanisms, which could be influenced by AD‐related processes that act differently in males and females. Further studies in larger, well‐powered cohorts are needed to clarify these observations.

In this study, we evidenced a negative correlation between CSF sclerostin levels and Aβ42 that strengthened previous data on the positive impact of circulating sclerostin on brain Aβ deposition in cognitively normal elderly at high AD risk, patients with AD, and transgenic AD model mice.^15^, ^16^ As a novel finding, our stratified analysis demonstrated that CSF sclerostin increase was closely associated to brain Aβ deposition in prodromal AD (i.e., in MCI stage). This is particularly relevant because it suggests that sclerostin may contribute to early pathogenic processes in AD, rather than merely reflecting later‐stage disease severity, highlighting its potential role in the initial phases of amyloid pathology.

Recently, the molecular mechanisms linking sclerostin to Aβ production have been investigated.^15^ Indeed, it was shown that the ability of osteocyte‐derived sclerostin to accelerate Aβ production was due to the upregulation of β‐site amyloid precursor protein (APP)‐cleaving enzyme 1 (BACE1), a key enzyme for Aβ production.^15^

The positive correlation between CSF sclerostin and tau proteins, p‐tau and t‐tau, evidenced the link with tau pathology, another key pathological feature in the continuum of AD, and neurodegeneration, respectively. Despite correlation does not imply causation, it is noteworthy that the positive association between CSF sclerostin and tau proteins persisted specifically in dementia due to patients with AD but was not observed in those with MCI, suggesting that this relationship is dependent on disease stage rather than reflecting a general group effect. The association with p‐tau has been already described in old subjects at high risk of AD,^16^ but here it was demonstrated for the first time across the disease stages. Of note, we found similar trends in patients with MCI and AD; indeed, we noticed slightly higher p‐tau and t‐tau levels in patients with MCI and AD with higher CSF sclerostin concentrations. Although the role of sclerostin on neurofibrillary tangle formation is still not clear, it has been demonstrated that the suppression of Wnt‐β‐catenin pathway activates glycogen synthase kinase‐3β (GSK‐3β), a kinase that increases tau phosphorylation.^33^ Therefore, as Wnt‐β‐catenin pathway inhibitor, sclerostin could be also involved in neurofibrillary tangle formation promoting this process.

Importantly, CSF/serum albumin quotient (Q‐Alb) values did not differ significantly across SMC, MCI, and patients with dementia due to AD, indicating that major alterations in blood–brain barrier integrity were not present in our cohort. This finding suggests that the observed increases in CSF sclerostin levels in patients with MCI and AD are unlikely to be a consequence of non‐specific leakage due to barrier dysfunction. Rather, these results support the notion that elevated CSF sclerostin reflects disease‐related biological processes within the AD continuum, strengthening its potential relevance as a biomarker of early pathological changes.

To the best of our knowledge, our study investigated for the first time the association with clinical parameters of dementia severity evidencing a positive correlation and a positive trend with CDR‐GS and CDR‐SOB, respectively. On stratifying study participants by CDR‐GS, we noticed an increase in CSF sclerostin levels in patients with mild dementia. Although exploratory in nature, this finding suggests a potential association between sclerostin and early clinical stages of AD, warranting further investigation.

## 5 LIMITATIONS

The present study had some limitations. Despite the fact that in our analysis we have considered two demographic variables, such as age and sex, which are important in AD biomarker research^34^ and for their impact on sclerostin levels, there are several other factors that can influence sclerostin concentration, such as musculoskeletal status, chronic kidney disease, type 2 diabetes, and so on.^35^ First, sclerostin is mainly produced by osteocytes, therefore it is considered a relevant marker of the pool of mature osteocytes and reflects bone health.^36^ In this study, detailed information on patient musculoskeletal status, including the presence of bone and joint disorders (e.g., osteoporosis, rheumatoid arthritis, and osteoarthritis), which are common in aging populations, and/or the use of bone‐targeting therapies, was not available. Accordingly, further investigations incorporating comprehensive musculoskeletal profiling will be required to more rigorously assess the specificity and pathophysiological relevance of sclerostin in AD. In this context, the lack of a direct comparison to other osteokines (such as osteocalcin or lipocalin‐2), already studied for their involvement in AD pathogenesis,^9^, ^10^ further limits the ability to assess the uniqueness of sclerostin within the bone–brain axis. Thus, future studies that include parallel assessments of multiple osteokines could potentially contribute to a more nuanced interpretation of sclerostin's role within the bone–brain axis and help clarify whether its association with AD reflects a specific effect or a broader pattern of bone‐derived signaling.

Besides musculoskeletal status, in our future analysis, we will also need to evaluate the presence of concomitant diseases, such as type 2 diabetes and chronic kidney disease that also could influence serum sclerostin levels.^37^, ^38^ Second, given that this is a cross‐sectional study, it is not possible to establish a causal relationship between sclerostin and AD pathogenesis as assessed with biomarkers and clinical data. However, results seem to be a good starting point for future longitudinal and multicenter studies. Third, our results were obtained from CSF samples, which limits their immediate translational applicability due to the invasive nature of CSF collection. Moreover, the overlap of CSF sclerostin levels across disease stages represents an important limitation for the potential use of sclerostin as a standalone diagnostic biomarker, as it may result in a low negative predictive value. In addition, previous studies assessing bone‐derived markers in Parkinson's disease excluded sclerostin from the analysis due to its low detection rate.^39^ Taken together, these limitations related to CSF sclerostin suggest that further validation in more readily obtainable biological fluids, such as plasma or serum, will be warranted to greatly enhance the translational and clinical significance of sclerostin.

Finally, a further limitation of the present study is the lack of apolipoprotein E (*APOE*) genotyping. Given the well‐established role of the *APOE* ε4 allele in modulating amyloid deposition and tau pathology, we cannot exclude that the associations observed between CSF sclerostin levels and AD biomarkers may be influenced by *APOE*‐related genetic risk. Future studies incorporating genetic information will be needed to disentangle the contribution of bone‐derived factors from *APOE* ε4‐driven mechanisms in AD pathophysiology.

## 6 CONCLUSIONS

We have strengthened previous literature data on the association between sclerostin and brain Aβ deposition in early clinical AD. Furthermore, we have provided preliminary evidence for an association between sclerostin and tau pathology, as well as dementia severity, in the AD continuum. Overall, our findings, supporting the idea that osteocyte‐derived sclerostin could be a potential AD biomarker in the early phase of AD, are of great clinical significance for an earlier diagnosis of the disease. Moreover, beyond its potential role as an early diagnostic biomarker, sclerostin may also represent a novel and biologically plausible therapeutic target, provided that a pathogenetic role of sclerostin is demonstrated in future studies.

## CONFLICT OF INTEREST STATEMENT

The authors declare no conflicts of interest. Author disclosures are available in the supporting information.

## CONSENT STATEMENT

All participants enrolled in this study provided their written informed consent.

## Supporting information



Supporting information

Supporting information

Supporting information

Supporting information

Supporting information
