# Supplementary material for: Cerebrospinal fluid sclerostin levels in the early Alzheimer's disease stages
Source: Alzheimers Dement (Amst). 2026 Mar 11;18(1):e70297. doi: 10.1002/dad2.70297 (PMC12976974; doi:10.1002/dad2.70297)
Supplement: Supplementary file 1 — Supporting information [file DAD2-18-e70297-s005.docx]

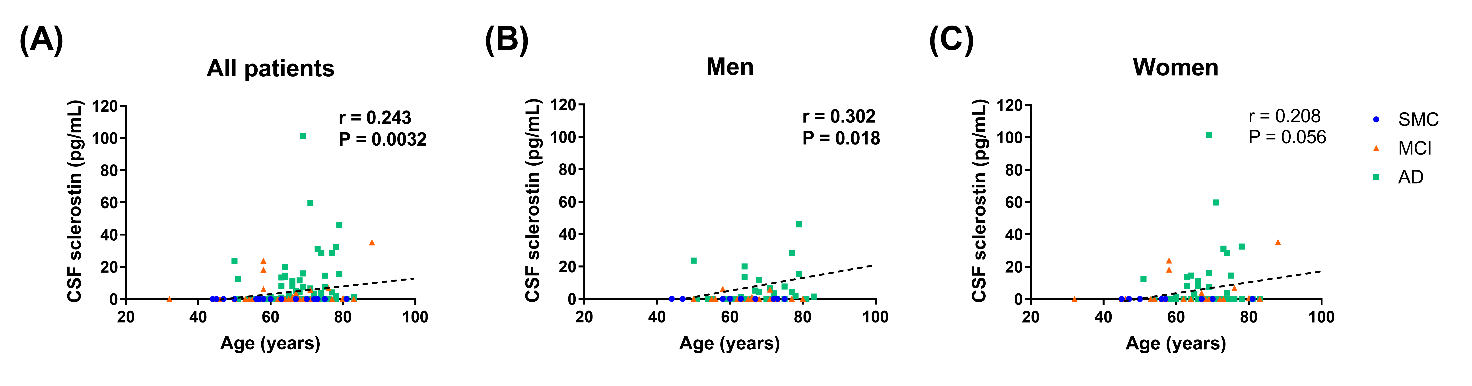


**Figure S1.** Correlation between CSF sclerostin and age. (A) CSF sclerostin levels positively correlated with age in all patient cohort, and (B) in male patients, while (C) a positive trend was observed in female patients. Dotted lines represent Spearman linear regressions (r and P values as indicated). Bold values highlight statistically significant correlations.

CSF, cerebrospinal fluid; SMC, subjective memory complaints; MCI, mild cognitive impairment; AD, dementia due to AD.
