# Supplementary material for: Cerebrospinal fluid sclerostin levels in the early Alzheimer's disease stages
Source: Alzheimers Dement (Amst). 2026 Mar 11;18(1):e70297. doi: 10.1002/dad2.70297 (PMC12976974; doi:10.1002/dad2.70297)
Supplement: Supplementary file 2 — Supporting information [file DAD2-18-e70297-s004.docx]

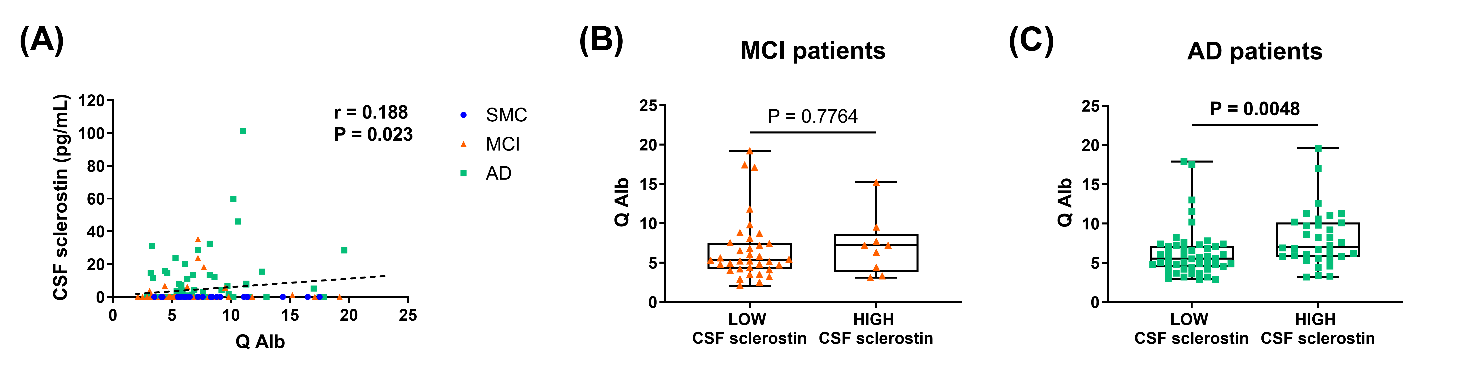


**Figure S2***.* Correlation of CSF sclerostin levels with Q-Alb, a marker of blood brain barrier integrity. (A) CSF sclerostin levels positively correlated with Q-Alb in all patient cohort. (B) Q-Alb value did not differ in MCI patients with HIGH and LOW CSF sclerostin levels, while (C) Q-Alb value was significantly increased in AD patients with HIGH CSF sclerostin.

In correlation plots, the dotted lines represent Spearman linear regressions (r and P values as indicated). For two group comparison, data are presented as box-and-whisker with median and interquartile ranges, from max to min, with all data points shown. Horizontal bars show the statistical analysis between groups (Student test or Mann-Whitney test, P < 0.05). Bold values highlight statistically significant results.

CSF, cerebrospinal fluid; SMC, subjective memory complaints; MCI, mild cognitive impairment; AD, dementia due to AD; Q-Alb, CSF/serum albumin quotient.
