# Supplementary material for: Cerebrospinal fluid sclerostin levels in the early Alzheimer's disease stages
Source: Alzheimers Dement (Amst). 2026 Mar 11;18(1):e70297. doi: 10.1002/dad2.70297 (PMC12976974; doi:10.1002/dad2.70297)
Supplement: Supplementary file 4 — Supporting information [file DAD2-18-e70297-s001.docx]

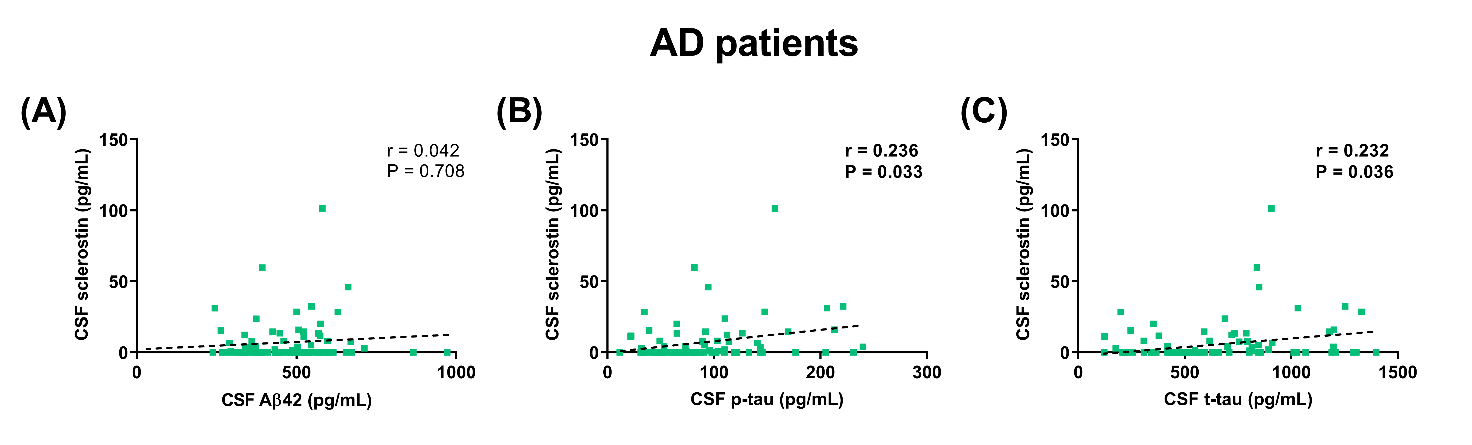


**Figure S4.** Correlation of CSF sclerostin levels and AD biomarkers in AD patients. (A) Sclerostin levels did not correlate with CSF Aβ42, while positive correlations were observed with (B) p-tau, and (C) t-tau. Dotted lines represent Spearman linear regressions (r and P values as indicated). Bold values highlight statistically significant correlations.

CSF, cerebrospinal fluid; AD, dementia due to Alzheimer’s disease.
